# Investigating the Impact of Two Major Programming Environments on the Accuracy of Deep Learning-Based Glioma Detection from MRI Images

**DOI:** 10.3390/diagnostics13040651

**Published:** 2023-02-09

**Authors:** Vadi Su Yilmaz, Metehan Akdag, Yaser Dalveren, Resat Ozgur Doruk, Ali Kara, Ahmet Soylu

**Affiliations:** 1 Department of Electrical and Electronics Engineering, Atilim University, Kizilcasar Mahallesi, Incek Golbasi, Ankara 06830, Turkey; 2 Fonet Information Technologies, Kizilirmak Mahallesi, Cukurambar Cankaya, Ankara 06520, Turkey; 3 Department of Electrical and Electronics Engineering, Gazi University, Eti Mahallesi, Yukselis Sokak, Maltepe, Ankara 06570, Turkey; 4 Department of Computer Science, OsloMet—Oslo Metropolitan University, Pilestredet 35, Oslo 0167, Norway

**Keywords:** brain tumor detection, glioma, deep learning, U-Net, V-Net, MATLAB, Python, performance assessment

## Abstract

Brain tumors have been the subject of research for many years. Brain tumors are typically classified into two main groups: benign and malignant tumors. The most common tumor type among malignant brain tumors is known as glioma. In the diagnosis of glioma, different imaging technologies could be used. Among these techniques, MRI is the most preferred imaging technology due to its high-resolution image data. However, the detection of gliomas from a huge set of MRI data could be challenging for the practitioners. In order to solve this concern, many Deep Learning (DL) models based on Convolutional Neural Networks (CNNs) have been proposed to be used in detecting glioma. However, understanding which CNN architecture would work efficiently under various conditions including development environment or programming aspects as well as performance analysis has not been studied so far. In this research work, therefore, the purpose is to investigate the impact of two major programming environments (namely, MATLAB and Python) on the accuracy of CNN-based glioma detection from Magnetic Resonance Imaging (MRI) images. To this end, experiments on the Brain Tumor Segmentation (BraTS) dataset (2016 and 2017) consisting of multiparametric magnetic MRI images are performed by implementing two popular CNN architectures, the three-dimensional (3D) U-Net and the V-Net in the programming environments. From the results, it is concluded that the use of Python with Google Colaboratory (Colab) might be highly useful in the implementation of CNN-based models for glioma detection. Moreover, the 3D U-Net model is found to perform better, attaining a high accuracy on the dataset. The authors believe that the results achieved from this study would provide useful information to the research community in their appropriate implementation of DL approaches for brain tumor detection.

## 1. Introduction

### 1.1. Preamble

Primary brain tumors refer to a heterogeneous group of tumors developing from various types of cells within the Central Nervous System (CNS) [1]. Primary brain tumors can be benign (non-malignant) or malignant. Non-malignant tumors grow slowly and do not spread to neighboring tissues, while malignant tumors expand rapidly and tend to invade other tissues [2]. According to the revised 2021 World Health Organization (WHO) classification of CNS tumors [3], brain tumors are mainly classified as: (a) Gliomas, glioneuronal tumors, and neuronal tumors; (b) Choroid plexus tumors; (c) Embryonal tumors; (d) Pineal tumors; (e) Cranial and paraspinal nerve tumors; (f) Meningiomas; (g) Mesenchymal, non-meningothelial tumors; (h) Melanocytic tumors; (i) Hematolymphoid tumors; (j) Germ cell tumors; (k) Tumors of the sellar region; and (l) metastases to the CNS. The Central Brain Tumor Registry of the United States (CBTRUS) reported that about 28.3% of all brain and other CNS tumors diagnosed in the United States between 2015 and 2019 were malignant, while 71.7% were non-malignant [4]. It is also reported that gliomas, including glioblastoma, pilocytic astrocytoma, oligodendroglioma, ependymoma, and a few rare histopathologies, were the most common malignant primary brain tumor type. Specifically, in the report, it is indicated that the most commonly occurring tumor was glioblastoma, which accounted for 14.2% of all tumors and 50.1% of all malignant tumors. According to the pathological and genetic properties, on the other hand, gliomas are classified by the WHO into four grades. Grades I and II are categorized as low-grade gliomas (LGG) such as pilocytic astrocytomas (WHO I), and diffuse low grade gliomas including diffuse astrocytomas and oligodendrogliomas (WHO II). Grades III and IV are categorized as high-grade gliomas (HGG), including anaplastic astrocytomas and anaplastic oligodendrogliomas (WHO III), and glioblastoma (WHO IV) [3,5]. Obviously, when compared to HGG, LGG are mostly considered as less-threatening tumors [6].

Diagnosis of gliomas at early stages is strictly necessary for a patients’ survival. For diagnosing tumors, the radiologists use different types of procedures or technologies such as biopsy, cerebrospinal fluid analysis, and medical imaging. However, with the rapid developments of computer science, imaging technologies have become more popular due to their accuracy and low risk to patients. Today, three imaging technologies such as X-rays, computed tomography (CT), positron emission tomography (PET), and magnetic resonance imaging (MRI), are commonly used in the diagnosis of brain tumors. Among them, MRI is the most preferred imaging technology because of its high-resolution image data that are useful for the detection and classification of tumors. However, the identification of tumors from MRI data is not an easy task for the experts, as they need to put forth significant effort in order to provide a diagnosis in a limited time. Therefore, automated diagnostic systems based on machine learning (ML) approaches have started to be used [7]. ML is mainly aimed at classifying tumors into the specific classes, such as tumor substructure, tumor/non-tumor, or benign/malignant tumor. 

Various ML methods based on particular classifiers such as Support Vector Machine (SVM) [8,9,10], Extreme Learning Machine-Improved Particle Swarm Optimization (ELM-IPSO) [11], K-Nearest Neighbors (KNN) [12], Artificial Neural Networks (ANN) [13], and Random Forest (RF) [14,15] are used for glioma detection in the literature. However, it is worth noting that such traditional ML methods apply pre-designed feature extraction from the MRI data. Evidently, pre-selection of features might adversely affect the performance of these traditional ML methods [16]. In order to resolve this concern, deep learning (DL) approaches have gained increased attention from many researchers [17]. One of the most important advantages over traditional ML methods is that hand-crafted features are not required in the implementation of DL approaches. Even complicated patterns can be discriminated by automatic feature learning. This enables practitioners to generate faster and better insights from large MRI datasets.

In addition to glioma detection, the use of DL approaches is also very popular in real-life applications in different fields, such as groundwater level prediction [18], nonlinear distributed thermal processes [19], or even the aquaculture and fishery industry [20], due to its easier implementation and higher computational efficiency. Many studies have been conducted to propose DL approaches to be implemented for efficient detection and classification of brain tumors as glioma, meningioma, or pituitary tumors from MRI data [21,22,23,24,25,26,27,28,29,30,31,32,33]. DL approaches based on Convolutional Neural Networks (CNNs) have attracted much interest, as they require minimum preprocessing [34,35,36,37,38,39,40]. However, it is very important to understand which CNN architecture would work well for detection and classification of brain tumors under various conditions including the development environment or programming aspects. Until now, two high-level programming environments, namely MATLAB and Python, have been commonly preferred in the literature to implement the DL approaches for the detection of brain tumors. Therefore, it is necessary to investigate the impact of these programming environments on the accuracy of CNN-based brain tumor detection.

### 1.2. Related Works and Research Gaps

High-level programming environments such as MATLAB, Python, R, Scala, Julia, or Java are able to empower scientist and engineers in order to implement their ideas in a faster way. This is due to the fact that the computational difficulties of low-level programming languages, such as C, C++, or FORTRAN, can be relaxed by high-level programming environments, which allow users to deal with mathematically heavy problems by means of minimal programming. Among high-level programming environments, the use of MATLAB and Python is mostly preferred in DL implementations due to their easy learning, simplicity of use, and adaptability features.

The performances of MATLAB and Python compared to each other has been a subject of debate over the years. In this context, the performance of MATLAB has been comparatively assessed with Python for different applications in various fields [41,42,43,44,45,46]. In [41], the aim was to offer a well-structured teaching language that enabled engineering students to quickly express their algorithms. In [42], the comparisons were made in terms of execution times for a macroeconomic application. The study presented in [43] was conducted for researchers in the field of economics in order to offer a best programming environment suited to their own purpose. In [44], the performance of the programming environments in a bioinformatics application were compared in terms of their memory requirement and ease of integration. In [45], the runtimes of the programming environments were compared for complex number calculations used in electromagnetic applications. In [46], accuracies of the programming environments for the computation of a robotic arm end effector matrix were compared.

Motivated by the aforementioned discussion, there have been no studies presented that focus on the efficiency of MATLAB and Python on CNN-based brain tumor detection from MRI data. This, in fact, indicates a significant research gap that might be critical for many practitioners in their appropriate implementation of DL approaches for brain tumor detection.

### 1.3. Purpose and Contributions

This research work aims to investigate the impact of two major programming environments, namely MATLAB and Python, on the accuracy of CNN-based brain tumor detection, particularly glioma, from a well-known MRI dataset. For this purpose, extensive experiments on Brain Tumor Segmentation (BraTS) dataset are performed by implementing two popular and simple CNN architectures: a standard three-dimensional (3D) U-Net [47] and V-Net [48] in MATLAB and Python. In the experiments, due to its larger size and higher resolution, the BraTS 2016 and 2017 dataset, consisting of multiparametric-magnetic MRI from patients diagnosed with either HGG or LGG, was used [49]. Experimental results show that using Python with Google Colaboratory (Colab) offers significant advantages in terms of training time and accuracy over MATLAB. Results also indicate that the 3D U-Net model has higher accuracy in comparison to the V-Net model on the used dataset. Thus, it is believed that the comparative study provided in this paper would attract the research community working in the area of brain tumor detection from MRI images, and help them to explore some future research directions as well as applicability of the models to BraTS dataset. The main contributions of this study can be summarized as follows:

(a) This is the first study that comparatively assesses the impact of two major programming environments on the accuracy of CNN-based glioma detection.
(b) Glioma detection performances of two popular CNN architectures, namely 3D U-Net and V-Net, are compared using the BraTS dataset (2016 and 2017) for the first time in the literature.

The rest of the article is organized as follows. In Section 2, an overview of MATLAB and Python for DL implementations is provided. Then, in Section 3, the details of the experiments performed within the context of this study are presented. This is followed by Section 4, where experimental results are presented. Further discussions and future research directions are addressed in Section 5 and Section 6, respectively. Finally, the article is concluded in Section 7.

## 2. Overview of MATLAB and Python for Deep Learning

In this section, a brief discussion on the use of two major programming environments in DL implementations is presented to provide a better understanding before describing the experiments and results.

It is widely known that DL is a branch of ML, which teaches computers to learn from experience. In DL, neural networks combining nonlinear processing layers are used to learn useful features from the data. The effectiveness of DL models in object classification has been thoroughly discussed in the literature. However, the efficiency of MATLAB and Python in DL model implementations is still under debate by the research communities.

MATLAB is a popular high-level programming language often employed both in industry and research for numerical computations [50]. It is very mature and widespread among the scientific community as well as practitioners, with its user-friendly toolboxes and libraries for DL implementations. It supports Open Neural Network Exchange (ONNX), which is an open standard that defines common file format and set of operators to represent DL models in various frameworks. It is interoperable with Python, which empowers researchers to use MATLAB and Python together. Moreover, it is easy to preprocess datasets with domain-specific applications for various types of data. In other words, it is possible to check and fix problems in order to build or modify complex network architectures for transfer learning before the training process. It is also possible to produce code supporting optimized libraries such as the ARM Compute Library, NVIDIA TensorRT, and Intel Math Kernel Library for Deep Neural Networks (MKL-DNN). This multi-program deployment feature significantly improves its performance. Furthermore, MATLAB Deep Learning Toolbox provides a framework in order to design and implement DL networks or models. For instance, Long Short-Term Memory (LSTM) networks and CNNs can be easily applied by the users. Its applications enable users to update the network architectures, to check the progress in preparation, to visualize the layer activations, and to monitor training progress graphically. For training on a modest dataset, it is possible to operate transfer learning through the pre-trained network models or models trained with libraries such as Keras, Caffe, and TensorFlow. For training on larger datasets, the process can be boosted on a single or multiple graphics processing units (GPUs) with Parallel Computing Toolbox. In addition, the training process can be scaled up to clusters and clouds, such as Amazon Elastic Compute Cloud (EC2) GPU instances, and NVDIA GPU Cloud with MATLAB Parallel Server. Although MATLAB has many advantages for DL, not being open source might be considered as an important drawback. MATLAB is proprietary software, and it is expensive because of its commercial license. This, in fact, limits the flexibility of a professional programmer. In addition, the use of NVDIA GPU Cloud and Amazon EC2 instances is not free, which can be considered as another drawback in the training of larger datasets.

Python, on the other hand, is another high-level language that is increasingly used in various sectors [51]. It is open-source, and it has a rich ecosystem from which a variety of packages and libraries can be downloaded and installed for different tasks and purposes. It has very easy syntax and commands; therefore, it appeals to many programmers as it is very easy to learn. This makes it easier to build models for DL implementations. Since it is a general-purpose language, a set of complex DL tasks can be accomplished and prototypes can be easily built in order to test a product for DL purposes. It has an extensive set of libraries that are widely used for DL, such as PyTorch, Keras, Tensorflow, Caffe, and others. It also offers a variety of visualization tools, namely Matplotlib, seaborn and gplot, for better understanding of data. It could be a good choice for DL, as it offers flexible programming which enables the programmers to select the programming styles, including imperative style, functional style, object-oriented style, and procedural style. Moreover, Google Colab can be used to execute arbitrary python code for DL implementations. Colab provides free access to utilize computing resources including GPUs. Hence, when DL implementation issues such as the storage of dataset and GPU in DL implementations are concerned, Python enables users to use free high-performance GPUs and open cloud environments. This can be considered as an important advantage for training large datasets.

It is surely beyond doubt that the interest in the use of Python for DL is increasing day by day, whereas the benefits provided by MATLAB should not be ignored. However, it is very important to note that deciding on the right programming environment for DL implementations depends on the user’s perceptions and expectations. Thus, it might be better to use MATLAB and Python interactively for utilizing the abilities of each environment. We believe that this hybrid approach could be expected to be more widespread among the researchers in the near future.

## 3. Experiments

### 3.1. Dataset

Several open source datasets such as BraTS [52], Harvard [53], RIDER (Reference Database to Evaluate Response) [54], and ISLES (Ischemic Stroke Lesion Segmentation) [55] have been used in brain tumor analysis. However, the BraTS datasets, which are an open source repository of multi-institutional radiological data, are mostly used by the researchers in ML competitions (challenges) in order to optimize or evaluate their proposed models each year, due to the fact that BraTS datasets are the most challenging MRI datasets [56]. 

**Figure 1:**
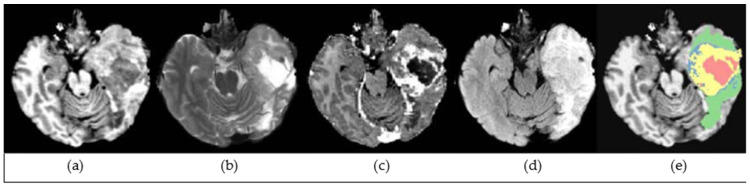
A sample of the dataset: (**a**) T1w (T1-weighted); (**b**) T2w (T2-weighted); (**c**) T1gd (T1-weighted with gadolinium contrast enhancement); (**d**) FLAIR (Fluid Attenuated Inversion Recovery); (**e**) Ground truth.

The brain tumor dataset utilized in the experiments is a volumetric medical image dataset, which is a subset of the data (Task 1) used in the 2016 and 2017 BraTS challenges [57,58]. This dataset was selected to be used in the experiments, as it is created for the challenge of locating the complex and heterogeneously-located targets while other BraTS datasets are created for different tasks [49]. The dataset consists of 484 multimodal multisite MRI images from patients diagnosed with either HGG or LGG. Each sample has four modalities, such as FLAIR (Fluid-Attenuated Inversion Recovery), T1w (T1-weighted), T1gd (T1-weighted with gadolinium contrast enhancement), T2w (T2-weighted), with ground truth labels, namely background, enhancing and non-enhancing tumor, and edema segmentations. Each image has 240 *×* 240 *×* 155 volume size, and has uniform, 1 mm^3^, voxel resolution. A sample of the dataset before preprocessing is shown in Figure 1 (adopted from [59]).

### 3.2. Deep Learning Approaches Used in the Experiments

In the literature, various DL approaches based on CNN architectures such as 3DConvNet [60], ResNet [61], DenseNet [62], GoogleNet [63], AlexNet [37], U-Net [64], V-Net [65] and VGG16 [66] have been used in MRI-based glioma detection, classification, segmentation, and grading. In general, all of these architectures demonstrate a good performance. However, in this study, a 3D version of the original U-Net architecture, which is adopted from [47], and a standard V-Net architecture [44] were chosen to be used in the experiments. One of the reasons is that they comply with our inclusion criteria, which included simplicity, relevance with glioma detection, and applicability to a volumetric medical image dataset. It should be noted that although the 3D U-Net and V-Net have been already applied to similar or closely-relevant applications, their glioma detection performances have not been comparatively assessed on the BraTS dataset (2016 and 2017) yet. This, in fact, is the other reason for choosing the 3D U-Net and V-Net architectures to be used in the experiments. In the following subsections, these architectures are summarized in technical aspects.

#### 3.2.1. 3D U-Net

A standard (two-dimensional: 2D) U-Net architecture is mainly composed of three parts: (a) Encoder, (b) bottleneck, (c) decoder [67]. In the encoder part, the prospective region of the image is identified. There are 3 × 3 convolution layers with activation functions, and 2 × 2 max pooling layers where the feature maps are doubled. The bottleneck part of the architecture is a path that assembles the encoding and decoding path. The decoder part has transposed convolutional layers with concatenation, and 3 × 3 convolution layers with activation functions. 

For glioma segmentation and/or detection from MRI data, the use of DL approaches based on U-Net variants has been proposed in many works [47,64,68,69,70,71,72,73,74,75,76,77,78]. However, since a volumetric image dataset is used in this study, a standard U-Net architecture and its variants developed for 2D datasets become inapplicable. For a proper implementation, it needs to be advanced by changing all 2D operations with their 3D counterparts. Hence, basic 3D U-Net [47] and its many variants, such as residual symmetric 3D U-Net [79], 3D U-Net++ [80], Attention 3D U-Net [81], and Separable 3D U-Net (S3D-UNet) [82] have been proposed for brain tumor segmentation. In this study, basic 3D U-Net framework is adapted due to its simplicity. 

**Figure 2:**
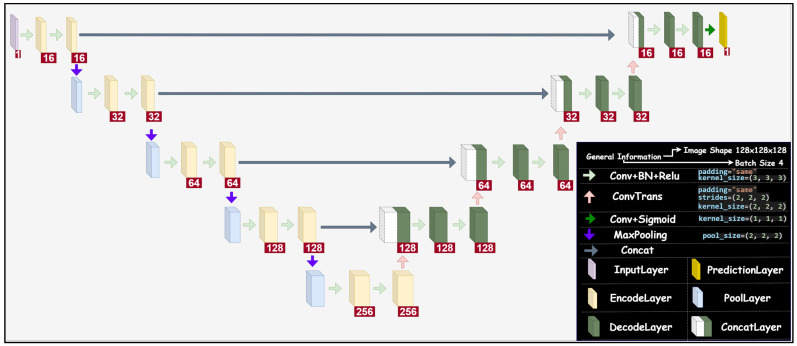
The network architecture of 3D U-Net used in this study.

The network architecture of basic 3D U-Net is shown in Figure 2. It should be noted that the numbers shown in the red boxes correspond to the number of filters. As shown in the figure, the input layer is followed by a convolutional layer, batch normalization, and a Rectified Linear Unit (ReLU) activation layer. Through the layers, padding is set to same, and the kernel size is kept constant at, 3 × 3 × 3. In addition, the max pooling layer is sized at 2 × 2 × 2 in the encoding path. In the decoding path, on the other hand, the number of filters is reduced by half after the transposed convolutional layers is applied with stride 2. A convolutional layer with a sigmoid activation function is then used for the segmentation of glioma. 

#### 3.2.2. V-Net

The standard V-Net architecture is a 3D CNN that is a modified version of standard U-Net for volumetric medical image segmentation [48]. In order to enhance the efficiency, various V-Net variants have been presented for brain tumor segmentation, such as Deep Supervised 3D Squeeze-and-Excitation V-Net (DSSE-V-Net) [65], Cascaded V-Net [83,84], Attention V-Net [85], and 3D AGSE-VNet [86]. For the sake of simplicity, the standard U-Net architecture is utilized in this study. 

In comparison to the 3D U-Net architecture, in a standard U-Net architecture, max pooling operations are replaced with convolutional layers which have a 5 × 5 × 5 kernel size in each stage. Moreover, throughout the network, Parametric ReLU (PReLu) non-linearities are applied. Another difference in comparison to U-Net is that the features extracted from the early stages of the left part of the network are forwarded to the right part of the network in order to gather fine-grained detail.

**Figure 3:**
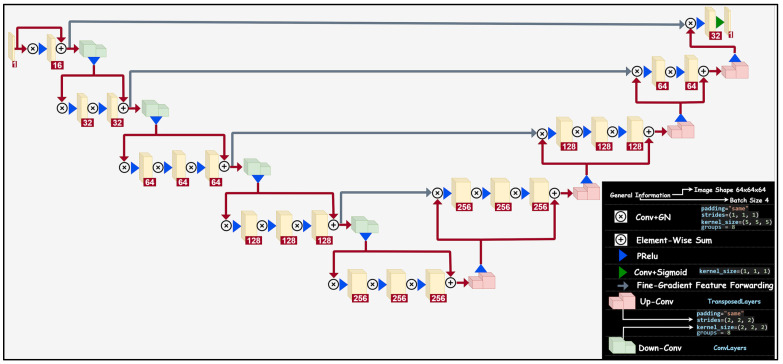
The network architecture of V-Net used in this study.

The network architecture of a standard V-Net is shown in Figure 3. In the left part of the network, there are four stages along with an input stage. The data resolution is reduced by means of convolutions with 2 × 2 × 2 kernels applied with stride 2. In the right part of the network, on the other hand, the very last convolutional layer has 1 × 1 × 1 kernel size, and produces outputs that are the same size as the input volume. After each stage, de(down)-convolution operation is applied, which is followed by convolutional layers with 1 × 1 × 1 strides. 

### 3.3. Preprocessing and Implementation Details

Before feeding the data into the U-Net and V-Net models, the data were preprocessed considering the networks’ architectures. In this context, the input dataset was resized to 128 × 128 × 128 dimensions for the 3D U-Net model, while it was resized to 64 × 64 × 64 dimensions for the V-Net model. The images in both datasets were then randomly flipped to reduce overfitting in the training of the models. Next, the dataset for each model is split up into the training set (400 images), the test set (55 images) and the validation set (29 images). 

The 3D U-Net and V-Net models described in Section 3.1 were implemented in Python and MATLAB. Google Colaboratory (Colab) was used as a development platform in order to implement the models in Python. In MATLAB implementations, on the other hand, the workstation equipped with NVIDIA Quadro RTX 5000 was used. Both 3D U-Net and V-Net models were trained on the same dataset introduced in the previous sub-section. In order to train the models, the Adam optimizer was used due to its faster computation time, and because it requires fewer parameters for tuning than other optimizers such as Gradient Descent, Adagrad, RMSProp, or AdaDelta. In addition, a smaller learning rate was decided to be used, which was set to 0.0003. Since it requires more training epochs in order to properly converge to a suboptimal solution, the epoch was set to 50. Moreover, in order to achieve the best performance of the workstation in terms of its computational power efficiency to process all images in parallel, batch size was varied and then determined to be set to 4. 

It is also worth noting that the input patch size was 4 × 128 × 128 × 128 to implement the 3D U-Net model, while it was 4 × 64 × 64 × 64 for the V-Net model, since there are four MRI modalities in the original dataset.

## 4. Results of the Experiments

Generally, the effectiveness of ML algorithms is evaluated under different goals, such as easy training, long lifetime, good performance, and rapid production. In order to compare the effectiveness of ML models or algorithms, development-based and production-based approaches can be used. While development-based approaches include statistical tests, loss functions and metrics, and learning curves (training and validation learning curves), production-based approaches include two basic parameters: time complexity and space complexity. 

On the other hand, since the main target of this study is to investigate the impact of two major programming environments on the accuracy of DL-based glioma detection, basic comparable parameters, such as learning curves, loss (accuracy) metrics, and training time, were chosen to evaluate the experimental results. Other comparable parameters can still be used; however, they are more applicable for comparing the effectiveness of DL algorithms comprehensively. Thus, firstly, the models were validated on the dataset, and their accuracies were recorded. Then, based on the comparative performances of the models, the effects of the programming environments on the results are assessed. 

**Figure 4:**
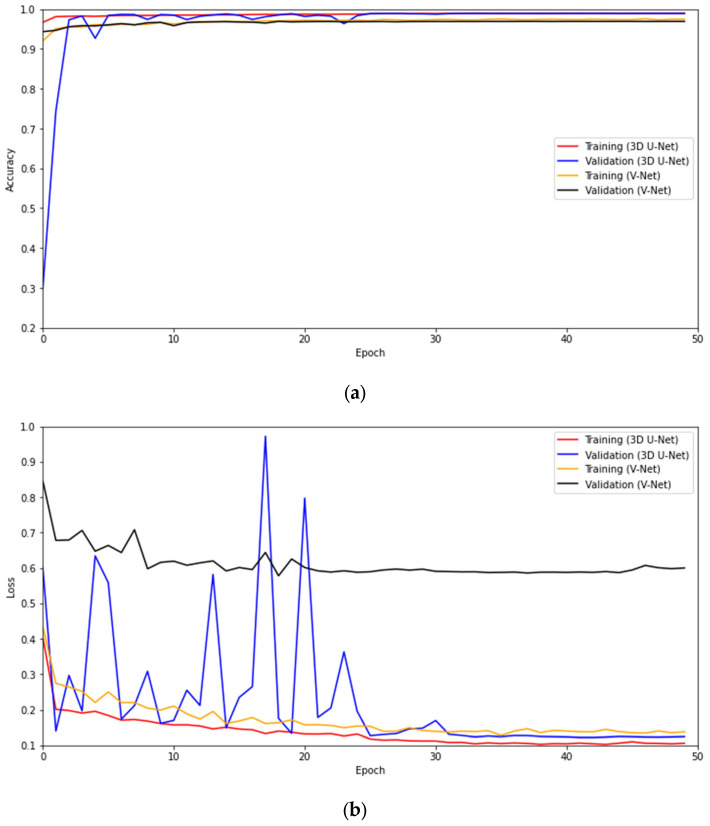
For the 3D U-Net and V-Net models implemented in Python: (**a**) Training and validation accuracy, (**b**) Training and validation loss.

The training and validation accuracy and loss of the 3D U-Net and V-Net models implemented in Python are shown in Figure 4a and Figure 4b, respectively. The 3D U-Net model achieves a training accuracy of 98.7% with a training loss of 13.7% and a validation accuracy of 96.6% with a validation loss of 36.2%. The test accuracy, moreover, is found to be 98.9% while the test loss is found to be 21.9%. On the other hand, the V-Net model provides a training accuracy of 96.9% with a training loss of 17.1% and a validation accuracy of 96.6% with a validation loss of 61.3%. Furthermore, the test accuracy of the model is found to be 96.9%, while the test loss is found to be 40.6%.

As for the 3D U-Net model implemented in MATLAB, the model achieves a training accuracy of 98.6% with a training loss of 26.4% and a validation accuracy of 97.7% with a validation loss of 37.6%. The test accuracy achieved by the model is 97.8%, and the test loss is 26.7%. The V-Net model implemented in MATLAB, on the other hand, achieves a training accuracy of 97.7 % with a training loss of 25.4% and a validation accuracy of 97.5% with a validation loss of 37.9%. Furthermore, the V-Net model achieves test accuracy of 97.2% while the test loss of 43.2%.

The comparative performances of the 3D U-Net and V-Net models implemented both in Python and MATLAB are summarized in Table 1. It is clear that the 3D U-Net model has higher accuracy when compared to the V-Net model regardless of the programming environment used in the experiments. Particularly, low training loss (17.1%) but high test loss (40.6%) obtained in Python experiments show that the V-Net model could be overfitting. This suggests that the V-Net model might not be applicable to the used BraTS dataset for glioma detection. Therefore, in order to assess the impact of programming environments on the detection accuracies, only the results achieved for 3D U-Net model are considered. 

From the results listed in Table 1, it can be seen that the accuracy of the 3D U-Net model is highly affected by the programming environment used in the experiments. The test accuracy of 98.9% achieved from Python is reduced to the accuracy of 97.8% when MATLAB is used. This is also the case for training loss of the models, which decreases from 26.4%, obtained in MATLAB, to 13.7%, obtained in Python.

Concerning the training time of the models, on the other hand, it took about 4 hours for training both the 3D U-Net and V-Net model in Python, while it took about 38 hours in MATLAB. The reason is that a free GPU supported and provided by Google Colab is much faster than the GPU of the workstation used to perform experiments in MATLAB. Moreover, during the implementation of models in Python, the data is retrieved from a free cloud service of Google Colab, whereas it is retrieved from MATLAB folders. Therefore, using Python with Google Colab offers a significant advantage over MATLAB in terms of training time.

## 5. Discussion

The results achieved from the experiments can be discussed under two main heads. One is the effect of programming environments on the accuracy of CNN-based models in glioma detection from MRI images. From the experimental results, it can be concluded that the detection accuracies of the models can be affected by the used programming environment. This can be clearly seen in Table 1, where the test accuracy of 3D U-Net obtained from Python is decreased by 1.1% when MATLAB is used. This is also the case for test loss, which is increased by 4.8% when MATLAB is used. On the other hand, when the training time is considered, Python has an important advantage over MATLAB due to Google Colab, with the native features available for any user such as faster GPUs and cloud data storage. In Table 1, it is clear that the training time takes longer when MATLAB is used. However, it is still possible to achieve higher training times with MATLAB, if a powerful workstation is available. 

The second head is the comparison of the effectiveness of 3D U-Net and V-Net models utilized for detecting glioma images (tumor and non-tumor) from BraTS 2016 and 2017 dataset. The experimental results show that the 3D U-Net model has higher accuracy than the V-Net model. However, its performance needs to be compared with the state-of-the-art in order to quantify its efficiency over other available models. In the literature, only a few studies that test DL based on the same dataset for detecting glioma images (tumor and non-tumor) [87,88]. In [87], the SoftMax layer is used with the central clustering algorithm for feature extraction. The extracted features are then used in a CNN-based algorithm. Results show that the proposed approach provides 96% accuracy. In [88], a data augmentation-based DL approach is proposed. By means of a ResNet-50 classifier, the experimental results show that the proposed approach achieves 91.08% accuracy. Therefore, the results obtained in the study offer that the 3D U-Net with 98.9% test accuracy has an ability to reach higher accuracies in glioma detection when compared to the approaches proposed in both [87] and [88]. 

## 6. Future Research Directions

Although this study addresses some major issues on DL developments, there are still important research opportunities. In fact, the authors are currently working on some specific aspects. First of all, some other programming environments have gained interest in recent years, such as R, Scala, Julia, and Java, and are planned to be used in DL implementations in order to obtain more comprehensive comparison results. In this way, it will be possible to provide a deeper insight regarding the programming environments to be used for implementing DL-based models in brain tumor detection.

In this study, among the CNN-based models, 3D U-Net and V-Net were chosen to be implemented in MATLAB and Python for brain tumor detection. Obviously, other well-known CNN-based models could have been used in the study. In the literature, however, glioma detection performances of 3D U-Net and V-Net on BraTS dataset (2016 and 2017) have not been compared yet. Therefore, the main motivation behind our choice was to compare their glioma detection performance for the first time in the literature. In this context, our second task is to conduct new experiments on the most up-to-date BraTS datasets by accounting for other well-known CNN-based models, in order to evaluate their glioma detection accuracies.

It is important to note that the objective of the detection process in this study is to identify whether glioma is present in MRI images or not. As mentioned earlier, the primary brain tumors are categorized as meningioma, pituitary, and glioma. Many studies presented in the literature focus on the identification or classification of the primary brain tumors from MRIs. Moreover, classifying glioma grades as LGG or HGG from MRI images has received great interest in the literature. Thus, as a third task, the current study will be directed toward examining the effects of programming environments on the classification of primary brain tumors or glioma grades.

Furthermore, it would be interesting to evaluate the experimental results from the clinician’s point of view, due to the fact that clinicians mostly need accurate and reliable results. This idea has therefore motivated us to involve clinicians in our future works. 

## 7. Conclusions

This work was devoted to examining the impact of two major programming environments, namely MATLAB and Python, on the accuracy of glioma detection using CNN-based DL models. For this purpose, experiments were performed in which two popular CNN architectures were implemented on a well-known BraTS dataset (2016 and 2017). According to the experimental results, the use of Python with Google Colab brings some benefits in comparison to MATLAB. More specifically, using Python might affect the accuracy of the models positively, and require less training time. On the other hand, when compared to the V-Net model, the 3D U-Net model demonstrates higher accuracy. This suggests that the V-Net model could not be applicable to BraTS datasets, and further modifications might be required to improve its accuracy.

In general, it is expected that the results obtained in this study might offer some insights on the most appropriate DL development environment for brain tumor detection. In the near future, the authors are planning to enrich the presented study by considering different datasets, DL models, and programming environments in order to provide more comprehensive results. 

## Figures and Tables

**Table 1:** Comparison of the models implemented in Python and MATLAB.

Metric	Python		MATLAB	
	3D U-Net	V-Net	3D U-Net	V-Net
Training Accuracy (%)	98.7	96.9	98.6	97.7
Training Loss (%)	13.7	17.1	26.4	25.4
Validation Accuracy (%)	96.6	96.6	97.7	97.5
Validation Loss (%)	36.2	61.3	37.6	37.9
Test Accuracy (%)	98.9	96.9	97.8	97.2
Test Loss (%)	21.9	40.6	26.7	43.2
Training Time (hr.)	~4	~4	~38	~38

## Data Availability

The data presented in this study are openly available at http://medicaldecathlon.com/, accessed on 19 December 2022.
